# Aneurysmal artery supplying pulmonary sequestration: successful combined approach

**DOI:** 10.1093/icvts/ivae079

**Published:** 2024-04-27

**Authors:** Antonio Giulio Napolitano, Eleonora Coviello, Gioele Simonte, Jacopo Vannucci

**Affiliations:** Thoracic Surgery Unit, Department of Surgical Sciences, Santa Maria della Misericordia Hospital, University of Perugia Medical School, Perugia, Italy; Thoracic Surgery Unit, Department of Surgical Sciences, Santa Maria della Misericordia Hospital, University of Perugia Medical School, Perugia, Italy; Vascular and Endovascular Surgery Unit, S. Maria Della Misericordia University Hospital, Perugia, Italy; Thoracic Surgery Unit, Department of Surgical Sciences, Santa Maria della Misericordia Hospital, University of Perugia Medical School, Perugia, Italy

**Keywords:** TEVAR, IPS, Lobectomy, VATS

## Abstract

A 62-year-old man was diagnosed with an asymptomatic intralobar pulmonary sequestration supplied by a short-neck proximal 57×25 mm aneurysmal artery originating from the mid-descending aorta. The patient underwent thoracic endovascular aneurysm repair; an aortic endograft was released to entirely restore the aberrant vessel. Then, pulmonary resection was performed. A triportal video-assisted left lower lobectomy was carried out. The thoracic endovascular aneurysm repair minimized the risk of bleeding and allowed a safe pulmonary resection with a minimally invasive approach.

## INTRODUCTION

Pulmonary sequestration is an uncommon congenital malformation of the lungs characterized by nonfunctional lung tissue receiving its blood supply from an aberrant systemic artery, defined as intralobar pulmonary sequestration (IPS). Surgical resection remains the primary definitive treatment [1]. The traditional approach is typically a postero-lateral thoracotomy, although video-assisted thoracic surgery (VATS) is being performed with increased frequency [2]. Very few cases of IPS supplied by an aneurysmal systemic artery have been reported, and only one another case of a hybrid combined approach has been described [3].

## CASE REPORT

**Figure 1:**
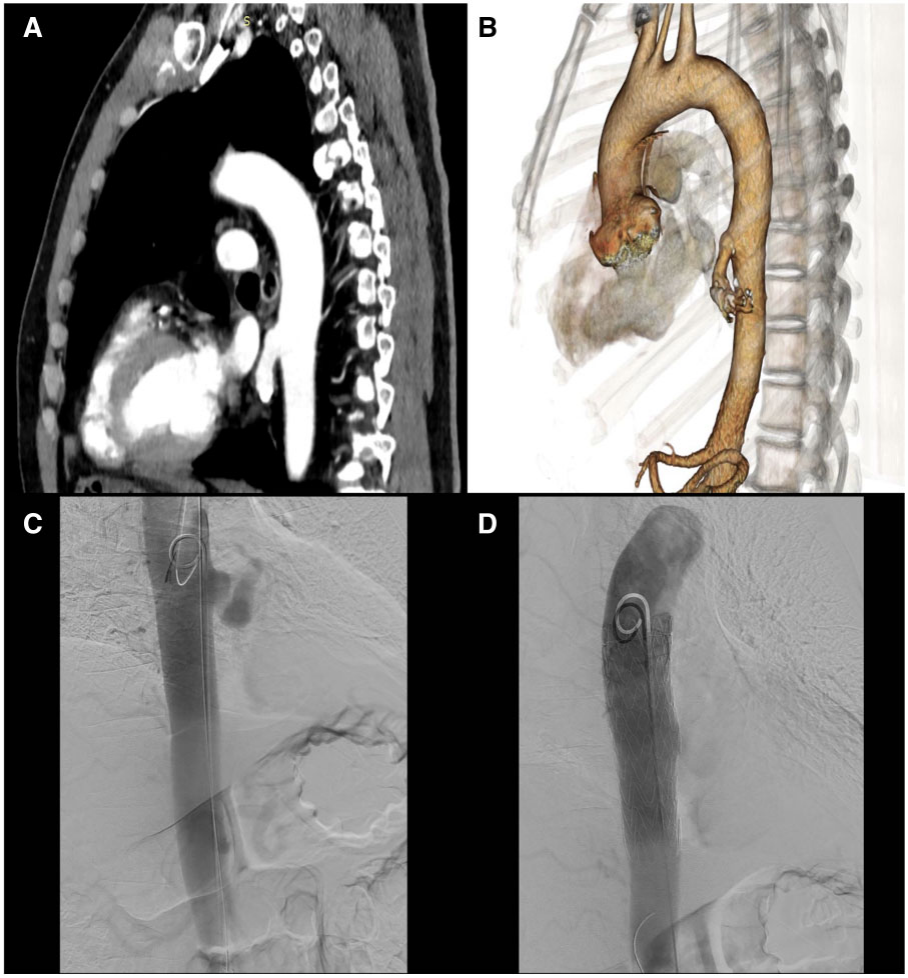
(**A**) Computed tomography scan of an aberrant aneurysm artery; (**B**) 3-dimensional computed tomography scan of a vascular reconstruction; (**C**) aberrant vessel as seen on an angiography examination; and (**D**) endograft angiographic check.

**Figure 2:**
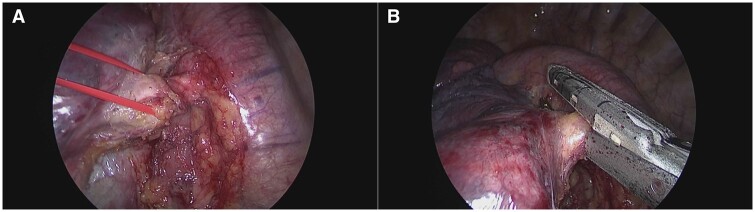
(**A**) Video-assisted thoracic surgery—isolation of an arterial vessel; (**B**) sectioning of an aberrant artery with a Vascular Endo Gia 30-mm stapler.

A 62-year-old man with a history of pneumonia underwent a computed tomography scan in July 2023 that showed an aneurysm of the aberrant artery, at its origin, with a 57-×25-mm vascular dilation. In addition, densitometric values of the left lower lobe parenchyma were higher than normal, suggesting a pulmonary sequestration (Fig. 1A). A transthoracic echocardiogram showed a moderate increase in the left ventricular filling pressure. The surgical indications were discussed in a multidisciplinary setting. After the multidisciplinary team viewed the computed tomography scan with a 3-dimensional reconstruction to accurately measure the vessel (Fig. 1B), the patient underwent thoracic endovascular aortic repair (TEVAR). After the pre-implantation of 2 Perclose haemostatic systems (Abbott Cardiovascular Laboratories, Santa Clara, CA), an 11-Fr introducer sheath was placed in the right common femoral artery. After administration of systemic heparinization, a J-standard guidewire and a JR4 catheter were advanced from the right access through the aortic arch, and the J-standard guidewire was exchanged with a thoracic Lunderquist guidewire (Cook Medical, Bloomington, IN, USA). From the left access, a J-standard guidewire and a pigtail catheter were advanced through the aortic arch. After predilatation of the right access with an 18 Fr Gore Dryseal introducer sheath (33 cm), a 22 Fr Gore Dryseal introducer sheath (33 cm) was placed (W. L. Gore Inc., Newark, DE, USA). A 34- to 100-mm Gore TAG thoracic endograft (W. L. Gore Inc.) was advanced with the support of a roadmap, positioned and then deployed in the descending thoracic aorta, completely covering the vessel vascularizing the pulmonary sequestration (Fig. 1C). Angiographic monitoring showed the patency and the correct expansion of the endograft (Fig. 1D). At the end of the procedure, distal pulses were bilaterally valid with no clinical and no surgery-related events. The next day, a left triportal VATS lobectomy was performed. Intraoperatively, we found chronic flogosis-induced adhesions between the oesophagus and the lower pulmonary vein. Once the oesophageal adhesions were released, the pulmonary ligament was dissected, and the lung was mobilized. The systemic aberrant arterial branch was clearly identified, isolated (Fig. 2A) and sutured with the 30-mm Vascular Endo Gia stapler (Medtronic, Minneapolis, MN, USA) (Fig. 2B). A lower lobectomy was performed. The postoperative course was uneventful, and the patient was discharged on postoperative day 5.

## DISCUSSION

Pulmonary sequestration is a rare congenital malformation in which the arterial supply usually comes from the descending thoracic aorta. Regarding any particular arterial supply, only a few cases of IPS have been reported to show an aneurysm of the aberrant artery. Surely, isolated endovascular repair of the anomalous arterial supply is possible, especially when no aneurysmal artery is present, but, traditionally, the definitive treatment of pulmonary sequestration is the ligation of the feeding vessel and lung resection to avoid recurrent infection and clinical evolution. Very few cases of an aneurysm of an anomalous systemic artery have been reported: In one of them, the patient underwent embolization with an Amplatzer Vascular Plug II (Abbott Vascular Laboratories, Santa Clara, CA, USA) to permanently occlude the aberrant systemic artery, thereby avoiding an operation [4]. Although this treatment is less invasive for the patient, it cannot definitively exclude future operations, and, in case an operation is necessary, it certainly would be technically more demanding. Furthermore, the persistence of abnormal lung tissue, although the vascular disease has been treated, does not elude the risk of infection. Bleeding from the feeding aneurysmal vessel is eventually a serious, life-threatening event for the patient. In our experience, another case of pulmonary sequestration supplied by a giant aneurysmal aortic branch was managed, and we decided to embolize it preoperatively to safely make the transthoracic resection after a postero-lateral thoracotomy right lower lobectomy [5]. This report helps support the combined TEVAR and VATS approach as a safe and feasible treatment for IPS associated with an aortic aneurysm [3]. Total exclusion/plugging of the aneurysmal vessel performed by TEVAR, without the use of coils or other devices, allows the use of mechanical sutures and aids the pulmonary resection with a decreased risk of bleeding.

## Data Availability

All relevant data are within the manuscript and its Supporting Information files.
